# Post Bellum Immigration

**Published:** 1918-12

**Authors:** 


### Post Bellum Immigration.

In a country which is probably 20% foreign by birth and half foreign in recent ancestry, it is an easy form of diplomacy to advocate unrestricted immigration. But such a view is neither sound citizenship nor in accordance with the views of the majority of the more or less foreign element. From the time of his debarcation, the interests of the immigrant are identical with those of every other resident of the country and it sometimes seems as if the most conservative attitude toward immigration is held by those who have been in the country but a short time relatively.

Many persons fail to realize the independence of the problems of immigration and of race prejudice. Let us illustrate from medical experience. Frequently, we encountered a case which, on close questioning, gives about the following history: a hearty luncheon at breakfast time, a dinner at luncheon, a banquet at dinner, a late supper, all sorts of dainties between meals, badly balanced diet, and mutually conflicting ingredients at meals. And after this history is dragged out of the patient he calmly says, "I think that pie, or pork, or ice cream"—as the case may be—"is bad for me." Let us turn to Uncle Sam's case. Up to 1820, there had been comparatively little immigration, except in a few areas, for over a hundred years. There are no statistics of immigration up to this date and, if there were, it would be difficult to analyse them with allowance for increase of population by annexation of large territories. Following this date, the immigration for each decade bore the following ratios to the population at the beginning of the decade: 1820-30, 1.54%; 1820-40, 4.659%; 1840-50, 8.22%; 1850-60, 10.82%; 1860-70, 7.56%; 1870-80, 7.293%; 1880-90, 10.46%; 1890-1900, 5,854%; 1900-10, 11.52%; and for the four years 1911-1914, 4.479%. It may be said in passing that while the foreign element in each decade would be augmented by propagation more than it would be diminished by death, one can not estimate the recent addition to American stock simply by adding these percentages together, in spite of the fact that they have increased probably more rapidly than the original stock. Without discussing the mathematic problem involved, it may merely be said that if anyone holds the contrary view, he can readily convince himself that it is incorrect by assuming that the present rate of immigration will continue for about 40 years more, when there will be the reductio ad absurdum of a population 105% foreign. However, the fact remains that there has been an enormous influx into this country from other nations. There is no disguising the fact that this has involved considerable trouble. But when Uncle Sam, or his spokesman, states calmly that the trouble lies with this or that European sub-race, he is making the same mistake as in the medical case cited. This statement requires only slight qualification since our entry into the war.

We are inclined to attribute very little importance to racial characteristics, exclusive of those of the five great divisions of mankind. Our conception of the term race, as applied to the European ancestry of most of us, is quite incorrect. Europe has been subjected to tides of migration even more complicated than that from Europe to America, and producing very much the same kind of mixture within a period which is, ethnically considered, not sufficiently remote to have resulted in any fixity of race corresponding to national designations. A simple and really scientific test of race prejudice is to note how far one can tell the nationality or ancestry of his associates, either from physical appearance or traits. Barring dialect and preservation of national customs in those very recently from Europe, he will make all sorts of mistakes, even assigning different racial attributes to members of the same family.

But freedom from race prejudice is quite another matter from advocacy of unrestricted immigration. It is beyond question that every race has its undesirable members, either physically or mentally. Even without regard to actual undesirability, it is not well for any country to have in its midst, any considerable number of individuals who are essentially foreign to it in language, habits, and prejudices, especially if these prejudices are against other ingredients of the population. Yet it should, in all fairness, be admitted that neither of these theoretic difficulties has produced the dire results in practice, that might have been anticipated.

But, the problem of finding a place and of securing, almost from the outset, a living for one person a year, added to each average group of 18 families is an economic problem which this country has not solved and which may well be regarded as unsolvable in a satisfactory way. During the present war, immigration has been slightly less than emigration. We shall have a chance to "catch up" on the immediate digestion of immigrants and the emphasis placed on the obvious advantages which our country presents at the present, will undoubtedly expedite ultimate assimilation. In particular, it will probably induce more permanent residents to become citizens, than before—and the number of persons who have hitherto enjoyed the privilege of life in this country without desiring to assume citizenship, is surprising. But, if as anticipated by many, this reduction of the essentially foreign element is followed by a tremendous rebound after the war, the condition will be even more serious than before.

One factor of special importance should be considered in this connection. Hitherto, this country has regarded itself and the immigrant as the only parties to the contract. At the close of the war, there will be a tendency to desert impoverished and wasted countries and to escape future military service. On the other hand, from the standpoint of the European countries there will be urgent need of people and especially of men. Even women will not be willingly spared since, in many European countries, they habitually engage in agriculture, manufacture and trade, to a greater extent than in America. There has already been some friction due to the conflicting claims of the country of birth and of naturalization or residence. Italy, we understand, extends this claim even to American born children of emigrants. Problems may arise as to the good faith of reservists who have not returned to their native countries, though for the most part, such return has been practically impossible. The military spirit and mobilization continuing after the actual cessation of hostilities may, not unreasonably, lead to the attempt to inforce claims on emigrants, at least diplomatically, which have hitherto been neglected or which have resulted merely in the arrest while visiting his native country of an occasional American resident or citizen. We feel strongly that even with regard to the ultimate status of the immigrant himself, it must be recognized that a third interested party exists—the country of his birth. Hence, we believe that all future immigration should involve the joint consent of this country and of the native country. The recognition of this principle will prevent various unpleasant contingencies. Even in countries which, from our standpoint—and that of most enlightened nations—have unduly oppressed a larger or smaller part of the population marked tendencies toward democratic ideals have already evolved and the way will be cleared after the war, for exerting influence to insure not merely the greater freedom and comfort of the few who can escape but of the vastly greater number who cannot emigrate. As a corollary to the proposition that all migration to this country should be approved by the native country, provision can be made for the more thorough inspection of prospective emigrants in Europe and, upon embarking, the emigrant can feel secure against exclusion at an American port, there will be no tragic disruption of families, no disappointed hopes at the last moment, no period of suspense, less opportunity for evasion of careful inspection.

